# Zebra rocks: compaction waves create ore deposits

**DOI:** 10.1038/s41598-017-14541-3

**Published:** 2017-10-27

**Authors:** Ulrich Kelka, Manolis Veveakis, Daniel Koehn, Nicolas Beaudoin

**Affiliations:** 1 School of Geographical and Earth Sciences, University of Glasgow, Glasgow, United Kingdom; 2 School of Petroleum Engineering, University of New South Wales, CSIRO Energy and Minerals Sector, Sydney, Australia

## Abstract

Nature has a range of distinct mechanisms that cause initially heterogeneous systems to break their symmetry and form patterns. One of these patterns is zebra dolomite that is frequently hosting economically important base metal mineralization. A consistent generic model for the genesis of these periodically banded rocks is still lacking. In this contribution, we present for the first time a fully consistent mathematical model for the genesis of the pattern by coupling the reactive fluid-solid system with hydromechanics. We show that visual banding develops at a given stress and host-rock permeability indicating that the wavelength and occurrence of the pattern may be predictable for natural settings. This finding offers the exciting possibility of estimating conditions of formation of known deposits as well as forecasting potential exploration targets.

## Introduction

Banded, striped or wave-like patterns are very common in natural systems and can develop spontaneously in chemical reactions. They are found on animal skins and shells^1^ as well as in a variety of fluids and solids. The genesis of such patterns is linked to the formation of waves, in a system far from equilibrium. For example, a reaction-diffusion process develops waves, where a fast reaction precipitates a band of a mineral phase while diffusion depletes the surroundings in the respective reactants. The repetition of this process leads to the formation of several bands with a distinct spacing, an example of so-called self-organization. One of the fundamental works that was published on stationary waves or bands, goes back to Alan Turing^2^ in 1952. He introduced a reaction-diffusion system in which a pattern (Turing pattern) develops as a result of instabilities in the underlying reaction.

The formation of stationary wave-like patterns is very common in geosystems^3^. Examples are compositional layering in igneous or metamorphic rocks, layering during sedimentation, chemical banding including the banded iron formation^4^, Liesegang rings on fracture surfaces^5^ and in ore deposits^6^, as well as layering in fault and shear zones^7^. The studies of such nonlinear dynamic systems in geosciences gave rise to the concept of geochemical self-organisation^8,9^ in which patterns develop spontaneously out of initially unordered systems. Recent advances include stress-induced effects producing not only compositional bands but also a layering in porosity^10,11^.

**Figure 1:**
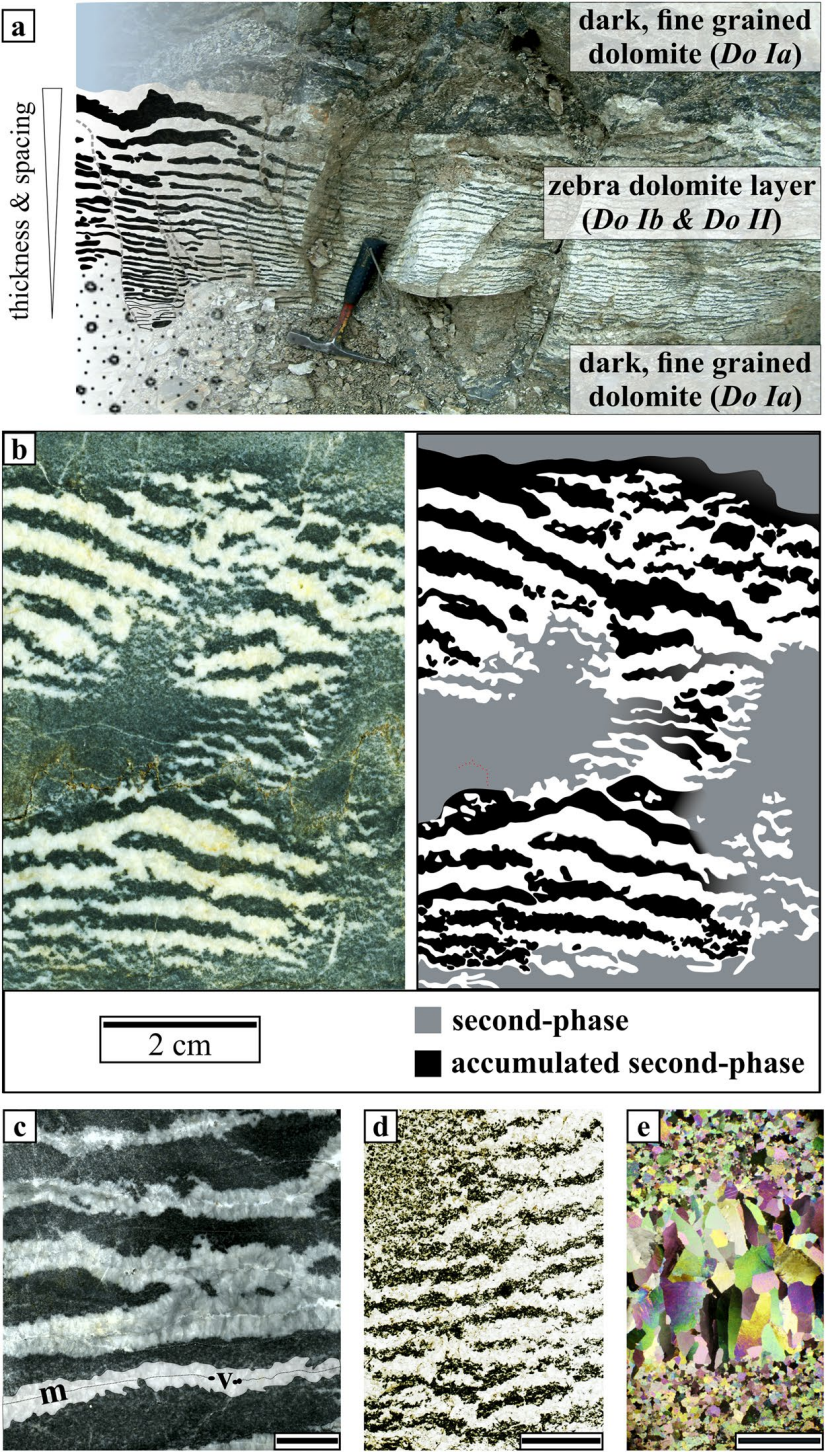
(**a**) Interpretative sketch and photograph of an outcrop of zebra dolomite layers at the San Vicente mine. Note that below and above the structure massive dark dolomite is visible. The width of the zebra dolomite layer is about 50 cm. (**b**) Scan of a polished hand specimen collected at the San Vicente mine and simplified sketch of the structure. **(c**) Scan of a polished hand specimen showing the detailed appearance of the zebra dolomite layers. The dark dolomite (*Do Ib*) alternates with light layers (*Do II*). In the central part of *Do II* a median line (*m*) with vugs (*v*) is observable (scale bar: 1 cm). (**d**) Scan of a thin section with thin narrow spaced zebra dolomite bands. In the upper left dark fine-grained dolomite is visible which does not show an impurity density as high as in the layers inside the zebra texture (scale bar: 1 cm). (**e**) Micrograph (XPL) of a single zebra dolomite layers with fine grained dolomite (*Do Ib*) below and above the coarse grained band (*Do II*) in the center (scale bar: 500 μm).

A striking example of waves in rocks is the zebra texture, a pattern that can be found in a variety of rock types ranging from claystones^12^, siderite-^13,14^ and sphalerite mineralization^15^ to hydrothermal dolomite formations^15–23^. In this work, we focus on the latter case, in which the texture of the periodic banded dolomites (zebra dolomites) consists of alternating dark and light bands (Fig. 1). The zebra texture in dolostones is frequently associated with base metal deposits of the Mississippi Valley-Type (MVT)^24^, whereby the banding predates the ore precipitation^25,26^.

What the underlying processes of the pattern formation in zebra dolomites are, is still debatable. Hypothesis on their genesis vary from the development of a fracture network^18^; opening of bedding/cleavage planes^27^; the development of a near-horizontal set of microfractures, along which dolomite precipitates^25^; pre-existing sedimentary partings^21^; sedimentary structures such as corals^25^; displacive vein growth^19,28^ or a form of geochemical self-organization^16^. In most of the areas where zebra dolomites are found, implications for an over-pressurized hydrothermal system^25–27^ can be observed, and zebra layers frequently form parallel or at a low angle to the bedding/foliation^18,22,27^.

**Figure 2:**
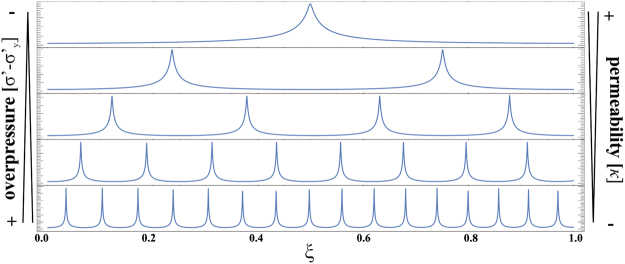
Response of the numerical solution of equation 3 to different overpressure (*σ′−σ′*_*y*_) and permeability (*κ*) at the scaled position (ξ). With increasing depth, the overpressure rise and the permeability will decrease. However, upwelling fluids that are confined within the host-dolomite by an overlying impermeable layer (e.g. shale cap) could as well generate the overpressure. Such structures are considered to indicate good potential for MVT-deposits^56^.

The purpose of this communication is to present a physically and chemically coherent model of zebra formation in a stressed sedimentary basin with evolving fluid pressure. Our model shows that the formation of rhythmic banded dolomites is the result of compaction instabilities that arise during a reaction-diffusion process in a system under applied stress, and that these instabilities can be mathematically described by periodic waves (cnoidal waves, see Fig. 2). Our 1D-model merges all existing hypothesis, and explains the typical structural features of the zebra texture in dolomites. Moreover, it is able to predict under what conditions they form.

### Zebras in mineralized dolostones

The MVT is a mineralization type that is typically hosted in carbonate formations of sedimentary basins that often contain dolomite^29^. While the lithology is relatively consistent, the orogenic type in which these deposits form varies (collisional, Andean or transpressional)^30^. The tectonic regime is therefore unlikely to be a first order control of MVT mineralization, or the zebra pattern formation. However, there is a spatial and temporal relation to orogenic foreland basin development reported for the mineralization^31^.

Field observations and the analysis of hand specimens show that a high variation in spacing and thickness of the bands exists on the outcrop scale. The distance between the centres of two light layers can vary between 2 mm to 10 cm (Fig. 1a). Laterally, the bands can extend as far as tens of meters and they can merge forming dislocation or cross-bedding like patterns. The bands also exist as isolated patches of layers confined by uniform dark dolomite (Fig. 1a). The dark matrix dolomite (*Do Ia*) is thought of originating from the replacement of the initial limestone^16,17,19,20,28^ and can be regarded as the host rock of the zebra dolomite. This dark dolomite is still lighter than dark dolomite bands (*Do Ib*) that are located in between the light bands (Fig. 1b,d). On the hand-specimen scale the light layers (*Do II*) display a median line (*m*) along which a vuggy porosity (*v*) is visible (Fig. 1c). In some areas, the centres of the light bands are filled with a late carbonate phase, which can be distinguished by the lighter colour of the material (Fig. 1c). The variability of the band spacing and thickness is assessable by comparing Fig. 1c,d: the spacing in Fig. 1c is about 1 cm in width whereas it is only about 2–3 mm in Fig. 1d. On the micro scale, a closer look at a single layer under cross-polarized light (Fig. 1e) reveals that the crystal’s size differs by several orders of magnitude between the fine-grained (*Do Ib*) and the coarse-grained layer (*Do II*). The fine-grained dolomite (in the dark bands) contains a large amount of impurities, which are partly clustered at the grain boundaries whereas the light layers are almost impurity free. In addition to that, the dolomite crystals in the coarse-grained light layers are elongated towards the central line, along which the void-filling carbonate cement is frequently observed^16,25,26,32^.

The samples analysed in this study were collected at the San Vicente mine (Peru) that represents one of the world’s largest ore deposits of the MVT^31^. The mine is located in the Subandean fold-and-thrust belt of the eastern Andean cordillera located about 300 km east of Lima. The hydrothermal mineralization consists primarily of the sulphide ore minerals galena and sphalerite. The ore bodies are strata-bound and hosted in Triassic/Jurassic platform carbonates (*Pucara Group*) in the western flank of the NW-SE striking *Pucara basin*. Faults likely provided pathways for the dolomitizing as well as mineralizing fluids^33,34^. The strata that host the mineralization are over-thrusted by younger plutonic rocks (*Tamra Granodiorite*). The setting of the San Vicente mine shows several of the typical features of environments hosting MVT mineralization^31^ and can therefore be regarded as a representative study area for the formation of banded dolomites and their relationship to mineralization.

### Theoretical description of pattern formation

In this section, we describe the development of the bands in the zebra dolomites by a phase separation process based on the *Cahn-Hilliard* and *Allen-Cahn* reaction-diffusion equations coupled with hydromechanics to mimic the pattern formation in a stressed sedimentary basin that undergoes diagenesis and builds up high fluid pressures. A reaction that can explain the virtually impurity-free dolomite in the light layers and the accumulation of impurities between these layers is the replacement of the primary dolomite by a secondary dolomite. While the dark dolomite (*Do Ia & Do 1b*) formed by the replacement of limestone will still contain impurities initially present in the rock, the replacement of the primary by the secondary dolomite (*Do II*) will segregate the impurities from the solid into the fluid (phase separation). Such a replacement reaction can be described as a process of coupled dissolution precipitation^35^ during which the impurity rich dolomite is locally dissolved and replaced by impurity free dolomite, thus leaving the impurities in the fluid. In the actual rock, this process takes place during grain growth in which impurities are collected in grain boundaries across which the dissolution-precipitation takes place and newly grown parts of grains become impurity free. In the natural samples, this process is indicated by the accumulation of impurities on grain boundaries in the dark layers. In addition to that, grains located at the transition between dark and light regions exhibit a sharp transition from an impurity rich nucleus within the dark bands to virtually impurity free crystals that grow towards the centre of the light layer (see Figure S4 in the supplementary material). The accumulation of impurities in the fluid can be regarded similar to industrial zone refining during which impurity-free crystalline materials are produced by a moving melting-freezing front^36^. That impurity rich layers in rocks can be the result of a similar mechanism had already been suggested by Krug *et al*.^13^. Effective cleansing and impurity redistribution by dissolution-precipitation can be achieved in rocks during grain growth as pointed out by Jessell *et al*.^37^. The basis of our model is a generic phase separation process that can be described as:

1 $$A{B}_{s}\rightleftharpoons {A}_{s}+{B}_{f}$$

In the case of the zebra dolomites AB_s_ represents the initial dark impurity rich dolomite (dolomite I) that is of replacive origin. The phase separation is driven by a fluid that accumulates impurities during dolomite-dolomite replacement (*B*_*f*_) and leaves behind an impurity-depleted dolomite phase (*A*_*s*_) after the reaction. The dark layers of the zebra texture are formed during the replacement of calcite by dolomite^19,20,25,26,28,32^, with an example shown in the supplementary material (Figure S4 in the supplementary material). The replacive origin of the primary impurity-rich dolomite (*AB*_*s*_) is shown by the preservation of initial sedimentary features (Ooids) in the dark bands^19^. The second dolomite generation of the light zebra bands (*A*_*s*_) appears as a coarse-crystalline impurity-free phase indicating an inverse correlation between final grain size and impurity density.

Typical MVT-fluids^19^ that are acidic, out of equilibrium with respect to the carbonate host-rock will enhance the recrystallization rates. This could explain why zebra dolomites are predominantly encountered within MVT districts. An Arrhenius law defines the mass production rate during grain coarsening. The rate of the reaction (*r*) can be written in a generic form as:

2 $$r={K}_{0}{e}^{-[\frac{E+PV}{RT}]}.$$

In this expression, *K*_0_ is a material specific rate constant, *E* is the activation energy of the process, *P* is the pressure related to the volume change process, *V* the activation volume of the reaction (which can be grain size dependent), *R* is the ideal gas constant and *T* is the temperature. Grain coarsening, as well as the dissolution of minerals in rocks, is a stress and grain size dependent process^38^. At elevated pressures, the dissolution rate will be higher than the precipitation rate and therefore we can consider the precipitation as the rate controlling mechanism of a coupled dissolution-precipitation reaction. The formulation of the model is based on a mixed mass balance expression for the solid-fluid system, derived from initially defined partial densities for the solid (*ρ*_*s*_) and the liquid (*ρ*_*f*_) phase respectively. All the derivations are detailed in the supplementary material. The model represents an extension of the classical compaction bands theory^39^ to a viscous non-linear rheology, similar to Veveakis *et al*.^40,41^ and Weinberg *et al*.^42^. We apply the equation of state on the expression of the mixed mass balance and assume isothermal conditions (*dT* = 0). We can further lower the complexity by reducing the problem to 1D and considering the steady-state limit (d/*dt* = 0). We then derive:

3 $$\frac{{\partial }^{2}P}{\partial {\xi }^{2}}+Pe{(\frac{\partial P}{\partial \xi })}^{2}-\lambda {P}^{m}+\mu {e}^{\alpha P}=0$$

4 $$Pe={\beta }_{2}{\sigma }_{0}$$

5 $$\lambda =\frac{{\mu }_{f}{\dot{\varepsilon }}_{0}{y}_{0}^{2}}{k{\sigma }_{0}}$$

6 $$\mu =\lambda \frac{{K}_{0}}{{\dot{\varepsilon }}_{0}}(\frac{{\rho }_{s}}{{\rho }_{f}}-1)(1-\varphi ){e}^{-(\frac{E-{\sigma }_{0}{\rm{\Delta }}V}{RT})}$$

7 $$\alpha =\frac{{\sigma }_{0}\Delta V\,}{RT}$$

**Figure 3:**
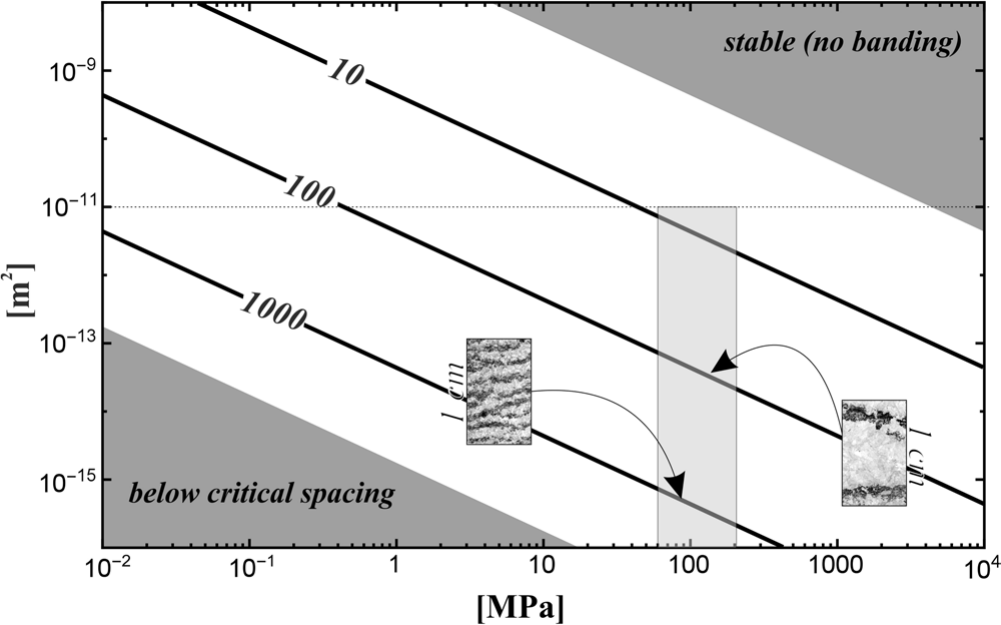
Log-log plot of permeability and over-pressure derived from equation 3 for a high saline brine at *T* = 80 °C, *μ*_*f*_ = 480 μPas^57^, *ρ*_*s*_ = 2.9 g/cm^3^, *ρ*_*f*_ = 1.26 g/cm^3^, *A* = 5∙10^−7^ mol m^−2^ s^−1^ (interpolation of experiments^58^), and the activation energy for dolomite precipitation *E* = 31.9 kcal/mol^59^. The lines indicate the numbers of zebra layers per m that can be related to the spacing of respective bands (images). The grey shaded area indicates the appropriate condition for buried dolomites. The dotted line indicates the maximum value for dolomite permeability^46^.

In the expression *P* denotes the normalised over-pressure $(P=\frac{\sigma \text{'}-\sigma {\text{'}}_{Y}}{{\sigma }_{0}})$, *ξ* is the normalized space ($\xi =\frac{y}{{y}_{0}}$, with *y*_0_ being the reference length scale) in a coordinate system moving with respect to the direction of compaction, *Pe* is the *Peclet* number and *m* is the stress exponent. The remaining identities *λ, μ* and *α* in equations 4–6 include the hydromechanical parameters (Table S1 in the supplementary material) whose values are stated in the caption of Fig. 3 and Table S2 (supplementary). Additional information on the development of equation 3 can be found in the supplementary materials.

The solution of expression 3 can include non-linear periodic waves given by an elliptic function. The power law exponent *m* in equation 3 dictates whether the solution is the *Jacobian*- (odd numbers) or the *Weierstrass*-function (even numbers)^11^. How both are related as elliptic functions was pointed out by Abramowitz *et al*.^42^. The wave peaks appear as equidistantly spaced stress singularities (elevated fluid pressure) where dissolution takes place, thus correlating with high permeability channels^40^ (for more details see Alevizos *et al*.^43^): The genesis of these hydromechanical instabilities represents the response of the solid-fluid system to compaction, whereas the formation of the instabilities does not need to appear simultaneous but is closely related to the solution of the wave equation. As discussed in the supplementary material, the solution is very weakly depending on the value of *Pe*, for the selected range of values of the parameters listed in Table S2 (supplementary). It therefore can be concluded, that the solution depends mainly on the parameters that include permeability and mean stress.

The dependence of the solution to equation 3 on stress (depth) and permeability is shown in Figs 2 and 3. The number of wave peaks grows with increasing depth and/or decreasing permeability. This consequently means that the amount of compaction instabilities (the number of light bands) or the amount of waves that occur in a fluid saturated rock, as described by equation 3, is a function of permeability and vertical stress.

### Scaling to field observations

The value of *λ* in equation 3 is critical as it includes the hydromechanical parameters, fluid viscosity and permeability, as well as the reference values for strain, stress and space. We further notice that *λ* has the highest impact on the solution of equation 3. In order to invert for the spacing between the light layers we fitted *λ* to the number of wave peaks (*NB*) for constant values of *μ* and *α*. By inserting appropriate values, it can be shown that the values of the latter two dimensionless parameters are relatively low and have only little influence on the result. We obtained a square root dependency for $NB=C\sqrt{\lambda }$ where *C* is a constant weakly depending on the value of *α* (see Figure S3 in the supplementary material). For typical values, we can accept $C=0.27$. This scaling for the number of bands can lead to a second scaling for their spacing (*h*), related to the compaction length^40^ $h={\delta }_{c}/C=\frac{1}{C}\sqrt{k\,\frac{{\sigma }_{0}}{{\mu }_{f}{\dot{\varepsilon }}_{0}}}$ found in the classical compaction bands theory^39^. With this value, it is possible to obtain a relationship to the spacing (*h*) and directly compare the prediction of our model to field data. The detailed description of the inversion routine can be accessed in the supplementary material section.

We applied fixed values of reference strain rate ($\dot{\varepsilon }$_0_), reference stress (*σ*_0_) and of the material parameter such as density, viscosity, dissolution rate, and activation energy (see Table S2 in the supplementary material). It is important to note that the experimental determination of the energy parameter can reach uncertainties of 200–300%^44^. As this value is included in the dimensionless parameter α we performed a sensitivity analysis to assess possible influences (Figure S3 in the supplementary material). For a combination of appropriate parameters, we obtain Fig. 3 where the light grey shaded area indicates realistic values of stress and permeability for buried dolomites. The upper and lower dark grey shaded areas indicate the regions in which no banding will be observed, either because equation 3 remains stable without periodic waves in the solution (stable area) or the distance between the layers is too narrow (critical spacing) to produce macroscopically visible bands as the spacing would be on the order of the grain size (~100 μm).

We can now quantify the relationship between permeability, overpressure and the density of bands. The overpressure in San Vicente is related to a burial depth of about 3 km which is the maximum burial depth of the strata hosting the zebra dolomites^17^. The yield stress can be defined as the point at which the material permanently deforms by 0.2%^45^. This state should be reached at relatively low stresses for dolomites and therefore the overpressure (*σ’-σ’*_*y*_) can be assumed to show only moderate fluctuation around the maximum pressure. The remaining critical parameter is the permeability, which is related to the porosity of the rock^46^. A variation of this parameter can explain the difference in band spacing and thickness (Fig. 1a). Additionally, the spatial localization of the zebra texture, which is observable in Fig. 1b,d, can be explained by local permeability contrasts.

The development of the compaction bands out of an initially heterogeneous rock is the primary stage of the pattern formation. The compaction bands have a higher permeability compared to the surrounding host rock and fluid flow as well as dissolution/precipitation processes will be focused inside these channels due to elevated fluid pressure. We hypothesise that, due to the development of the compaction instabilities, a local recrystallization takes place. During this process, the pre-existing impurities in the dolomite are washed out and accumulate outside of the channels (Fig. 1b). This is in good agreement with the findings of^25,27^ who interpreted the light bands as dissolution or recrystallization features that develop during focused fluid flow. However, our model does not require pulsed expulsion of fluids^27^ or the development of en-echelon fractures^22,25,27^. The grain growth process will affect the whole rock volume but will be favoured in areas of low impurity densities^47^, and therefore inside the compaction bands. Grain boundary migration in systems that are comprised of a layered distribution of second-phase material can produce structures which are very similar to the zebra dolomites^48^. We argue that a fracture can also develop in the central part of the impurity depleted coarsening layers (Fig. 1c), because the fluid pressure is at its highest in the central part of the compaction band and the breaking strength of the material decreases with increasing grain size^49^. This can quantitatively be described by the *Hall-Petch effect* ^50,51^, that gives a relationship between yield stress and crystal size. A developing crack is accompanied by a stress drop in the solid around the fracture. As grain growth is sensitive to stress, the crystals will tend to elongate towards this crack (Fig. 1e)^52^. Even without material failure, dissolution will occur in the central part of the coarse layer in response to the elevated fluid pressure. Such dissolution features were reported for several zebra dolomite occurances^16,25,26,32^ and crack-like structures filled with a late carbonate phase are shown in Fig. 1c.

### Towards an integrative model of zebra dolomite formation

**Figure 4:**
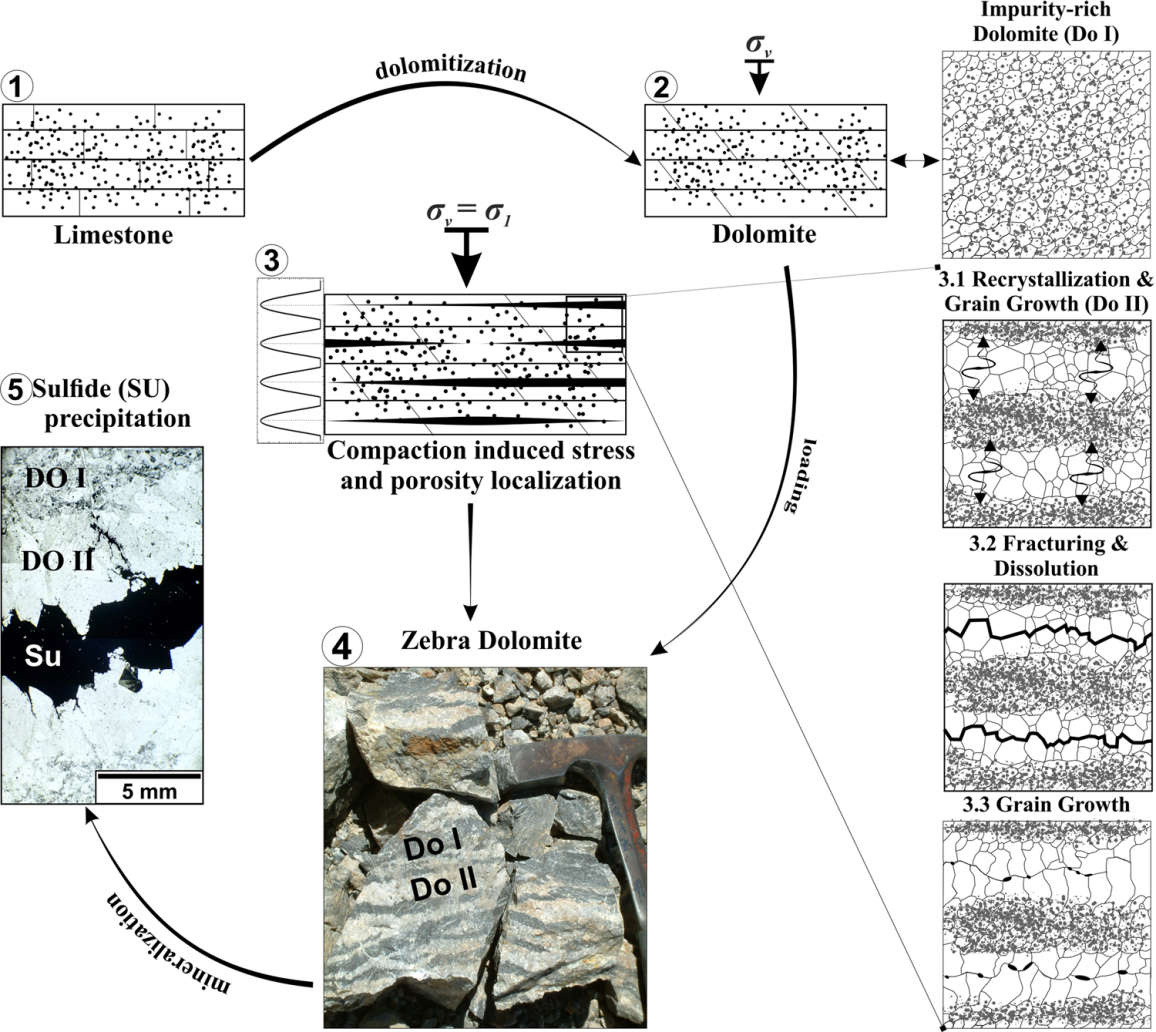
Conceptual model of zebra texture formation in dolomite involving the development of compaction instabilities leading to focused recrystallization and subsequent grain growth affected by second-phase particle densities. An impurity-rich limestone (1) is dolomitized by an infiltrating fluid (2). This fluid-saturated dolomite (*Do I*) undergoes compaction during burial, and instabilities evolve as soon as the critical values of permeability and loading are reached (3). The small plot next to sketch number 3 indicates how the solution of equation 3 is related to the localisation of high-porosity and high-pressure channels. Recrystallization is then focused in equidistant channels (*Do II*) whereas the second-phase is washed out and accumulated between the channels (3.1). The subsequent grain growth is now focused in the areas of low impurity densities. The high pressure then leads to fracturing in the high-pressure channels of the compaction bands as the yield stress is successively lowered during grain coarsening (*Hall-Petch relation* ^50,51^). The highest pressures occur in the centre of the compaction instabilities leading to fracturing and subsequent dissolution along the median line of the coarse grained layers (3.2). The grain growth continues whereas the grains now elongate towards the central line where stress is depleted due to the fracturing (3.3). The resulting texture is periodically layered (4). If a mineralizing fluid percolates into this structure, the sulphide (Su) will precipitate along the vuggy median line (5).

We presented a generic model of zebra dolomite formation (Fig. 4) based on the compaction band theory^11,40^ coupled with a reaction-diffusion model (see also Alevizos *et al*.^43^ for more details). In detail, our model is based on local dissolution-precipitation dynamics and, in contrast to other theories^19,20,28^, does not require displacive vein growth for the band’s equidistant spacing to occur. Our model also does not rely on initial sedimentary partings^21^ or the development of fracture networks^15^ (see supplementary material for further discussion).

We were able to show that the spacing of bands is a function of permeability and/or stress variations. Our approach is capable of integrating the findings of other works^16,18–20,22,25–28,46^ and it successfully explains all the specific features of the pattern. The layered distribution of impurities is caused by the focused recrystallization inside the compaction bands (Fig. 4.3) and the grain size variation is a direct result of the recrystallization^48^. The elongated shape of the crystals of the light layers is caused by the dissolution and/or fracturing along the central part of the layer (Fig. 4.3.2) as a function of high fluid pressures and large grain sizes. A vuggy porosity along the central line will remain and a late carbonate phase will precipitate in the median line (Fig. 4.3.3). The texture is important for ore mineralization. If a mineralizing fluid percolates into the structure, the sulphides will start precipitating along the median line (Fig. 4.5), which is often observed in the samples.

In line with Turing^2^ we propose a general process of pattern formation based on a reaction-diffusion equation but extended by hydromechanics. The basis of our model is the *Cahn-Hilliard* equation, where the process modelled is phase separation during which domains develop that contain a large amount of one of the two phases, a process that is often accompanied by pattern formation^53^. In our scenario, these accumulated phases are the impurities in the dark dolomite layers. In contrast to previously published theories on zebra dolomite formation we put forward a mathematical description of the reactive solid-fluid system coupled with hydromechanics. The predictions of our model can be scaled with field observation and we propose that it can represent a new tool for field-geologists to estimate rheological parameters such as permeability and stress. For now being solely a mathematical description, we state that our approach to pattern formation in dolomites represents one of nature’s general processes of producing periodic wave-like patterns in natural systems. Furthermore, the inversion routine applied in this communication demonstrates a relationship between the spacing of geological structures such as bands or layers and the fluid pressure and permeability during pattern formation. Having an access to these parameters is of high interest for scientific research as well as for mineral- or hydrocarbon exploration and extraction. In addition to that, the mathematical model predicts that the zebra layers form perpendicular to the main stress direction. It is therefore possible to determine the orientation of the stress-ellipsoid during the pattern formation as well. Thus, the model presented in this study represents the first step towards the development of a new tool for geologists that will help to assess paleo-fluid pressure, paleo-stress and paleo-permeability of geological formations hosting banded or layered structures.

Future exploration of mineral and energy resources will target deposits at greater depth. With increasing depth feedback mechanisms between mechanical compaction and chemical reaction rates will become more important. A complete understanding of the exact mechanisms that are active in such deeper environments will become crucial for successful prediction of deposit location and their exploration. In addition, the extraction of resources from deeper hosts may trigger feedback mechanisms that to date are not fully understood. Striking examples of such feedback mechanism are the production induced compaction in the Ekofisc oil field^54^ and the success of gas extraction in the Cooper Basin, Australia^55^.

## Electronic supplementary material

Zebra rocks: compaction waves create ore deposits-Supplementary information

Supplementary Dataset 1
