# Supplementary material for: Zebra rocks: compaction waves create ore deposits
Source: Sci Rep. 2017 Oct 27;7:14260. doi: 10.1038/s41598-017-14541-3 (PMC5660191; doi:10.1038/s41598-017-14541-3)
Supplement: Supplementary file 1 — Zebra rocks: compaction waves create ore deposits-Supplementary information [file 41598_2017_14541_MOESM1_ESM.doc]

**Zebra rocks: compaction waves create ore deposits**

**Supplementary information**

*Ulrich Kelka*^1^, Manolis Veveakis^2^, Daniel Koehn^1^ & Nicolas Beaudoin^1^*

*1 School of Geographical and Earth Sciences, University of Glasgow*, *United Kingdom*

*2 School of Petroleum Engineering, University of New South Wales, CSIRO Energy and Minerals Sector,*

*Australia*

*Corresponding author: [u.kelka.1@research.gla.ac.uk](mailto:u.kelka.1@research.gla.ac.uk)

[ukelka@gmx.com](mailto:ukelka@gmx.com)

Table S1: List of Symbols

|  | Strain rate |
| --- | --- |
| *A* | Rate constant |
| *d* | Grain size |
| *DoIa* | Dark replacement dolomite |
| *DoIb* | Dark layers within zebra texture |
| *DoII* | Light layers of zebra texture |
| *E* | Activation energy |
| *h* | Spacing between wave peaks |
| *j* | Production rate |
| *k* | Permeability |
| *K_0_* | Reaction rate |
| *P* | Scaled overpressure |
| *Pe* | Péclet number |
| *P_f_* | Fluid pressure |
| *Q_g_* | Activation energy |
| *r* | Reaction rate during grain coarsening |
| *R* | Ideal gas constant |
| *T* | Temperature |
| *v* | Velocity |
| *V* | Activation volume |
| *y* | Space |
| *y_0_* | Reference position |
| y_0_ | Reference position |
| *β_m_* | Compressibility |
| *ΔV* | Molar volume change |
| *μ_f_* | Fluid viscosity |
| ξ | Scaled position |
| *ρ_f_* | Density of the fluid |
| *ρ_s_* | Density of the solid |
| *σ_0_* | Reference stress |
| *σ_y_* | Yield stress |
| *φ* | Porosity |

**Derivation of equation 3 and inversion routine**

The complete description of a poro-mechanical system undergoing a dissolution-precipitation reaction as well as the solution techniques used can be found in Alevizos et al.^1^. Here we brief the main aspects of the model relevant for the present work.

We may define a mixture consisting of a solid and a fluid phase, which in turn are consisting of dolomite with enclosed impurities (solid phase) and a brine with fluidised impurities (fluid phase) respectively (see equations S1). The phase transition of such a mixture is adequately described by Eq. (1), whereas the densities of the different phases read:

(S1)

where *φ* is the porosity (volume of fluid phase over the total volume of the mixture, and *s* and *w* are the volume fractions of the impurities in the solid and fluid phases respectively. *ρ_1_* is the density of the solid phase and *ρ_2_* of the fluid phase.

We write mass balance for each phase in 1D ($\partial_{t}\rho_{a}+\partial_{y}\rho_{a}v_{y}^{a}=j_{a}$, where $a=1,2$), yielding:

Solid phase: (S1a)

Fluid phase: (S1b)

In these expressions $\rho_{s}=\left(1-s\right)\rho_{dol}+s\rho_{imp,s}$ , $\rho_{f}=\left(1-w\right)\rho_{brine}+w\rho_{imp,f}$, $\frac{D^{(a)}}{Dt}=\frac{\partial}{\partial t}+v_{y}^{(a)}\frac{\partial}{\partial y}$ is the material time derivative of the a-phase ($a=1,2$), and $-j_{1}=j_{2}=j$ the mass exchange rate. We may now add (S1a-b) to obtain the mass balance of the mixture:

Mixture: (S1c)

We may now assume an isothermal equation of state formulation for the densities of the fluid and solid phase, $\frac{1}{\rho_{a}}\frac{D^{(a)}\rho_{a}}{Dt}=\beta_{a}\frac{D^{(a)}p_{f}}{Dt}$ with $\beta_{a}$ the compressibility of the a-phase. We also accept Darcy’s law for the filter velocity and assume that the convective terms of the solid phase are negligible in comparison to the convective terms of the fluid phase (i.e. $v_{y}^{(2)}>>v_{y}^{(1)}$). This yields for the mixture’s mass balance equation (see also ^2^, ^1^):

(S2)

in this expression is the volumetric (vertical) strain rate, is the compressibility of the mixture, *k* the permeability, *μ_f_* the viscosity of the fluid and *j* the rate of the generation of fluid phase (brine and impurities) through the reaction of Eq. (1). This reaction is related to the specific surface area of the grains, and thus the evolution of the average grain size *d*, and we hereby assume a first order rate for simplicity in the mathematical treatment ^3^:

$j=\left(1-\phi\right)\rho_{s} K_{0} e^{-\frac{E-\sigma' \Delta V \left(d/d_{0}\right)}{RT}}$ (S3)

Note that in the above expression we have accounted for the sign of stress in compression, resulting to a negative contribution to the internal energy *E* by the term$\sigma' \Delta V \left(d/d_{0}\right)$. The reaction rate constant $K_{0}$ is a function of the specific surface area, expressed through the average grain size *d*:

(S4)

Where $d_{0}$ is a reference grain size and *A* is the rate constant for dolomite. The rate of the reaction (derived unit is 1/time) is therefore faster with increasing specific surface area, thus decreasing grain size. In this study we will consider the case of very fast grain size evolution with respect to mechanical deformation rate, thereby placing the grain size evolution at steady state, . Therefore, we deduce$d=d_{0}$.

Assuming *Terzaghi’s* stress decomposition ($\sigma=\sigma'-p_{f}$) and invoking the steady state approximation ($\partial/\partial t=0$), the final system of equations consists of momentum balance and mass balance for the mixture:

(S5)

For the rate of the mechanical deformation we accept that zebra bands are located in compacting environments, thereby assuming a nonlinear viscoplastic compaction law of the simple form , where $\sigma'_{Y}$ is the effective yield stress (see also^2^). Note that *σ’* and *σ’y* in this expression represent normalized identities respectively, whereas a value of *σ_0_* had been applied as reference value (see table S2). Keeping in mind Darcy’s law the system of equations (S5) reduces to a single equation for the effective stress:

(S6)

The system can be further reduced by introducing the dimensionless variables $\xi=\frac{y}{y_{0}} , P=\frac{\sigma'-\sigma'_{Y}}{\sigma_{0}}$ (where $y_{0} , \sigma_{0}$ are reference values of the space and stress, yet to be determined). Assuming small variations of background permeability and fluid viscosity, and that the yield stress does not vary in space, this yields:

$\frac{\partial^{2}P}{\partial\xi^{2}}+Pe\left(\frac{\partial P}{\partial\xi}\right)^{2}-\lambda P^{m}+\mu e^{\alpha P}=0$ (S7)

where

(S8)

thus reproducing equations 3-6 of the main text.

Equation (S7) is therefore the governing equation of our problem at hand, and needs to be solved for appropriate boundary conditions. For the problem of zebra rock formation, we abstract the mechanical problem into a very simple 1D compaction scenario, in a domain of height$y_{0}$. At the boundaries of this domain overstress $\sigma_{0}$ is applied, providing the following boundary conditions in real and dimensionless form

$\begin{matrix} \sigma'\left(y=0\right)=\sigma'\left(y=y_{0}\right)=\sigma'_{Y}+\sigma_{0}\Rightarrow\\ P\left(\xi=0\right)=P\left(\xi=1\right)=1 \end{matrix}$ (S9)

This system presents a bi-modal behaviour in its solution, obtaining a diffusive smooth solution for low values of λ, and admitting anti-diffusive solutions at high values of λ^1,2^. This response is invariant of the value of the exponent m, as both odd and even values have the same elliptical functions as solutions^1,2,4^ (Fig. S1). In this work we choose m = 3, since this is the power law exponent for most carbonate-rich rocks.

In the anti-diffusive case the solution presents periodic peaks as shown in Fig. 2 of the main text, with their spacing *h* obeying a square root scalling law: *h* = 4 δ_c_. In this expression *δ_c_* represents the compaction length that is defined is equation S10 below.

Figure S 1: Response of the solution of equation 3 to different stress exponents: (left) *m* = 2, λ = 100; (middle) m = 3, *λ* = 50; (centre) m = 4, *λ* = 30

From the spacing of the peaks it is possible to invert for values of permeability, strain and the associated overpressure for a respective number of peaks or bands. In order to obtain a convincing relationship between overpressure and permeability, the first step is to fit the number of wave peaks (*NB*) occurring in the solution of equation 3 to varying values of *λ* (figure S2a). Subsequently, a function of band intensity per meter (*ρ_bands_*) depending on the reference strain rate can be obtained via the compaction length:

. (S10)


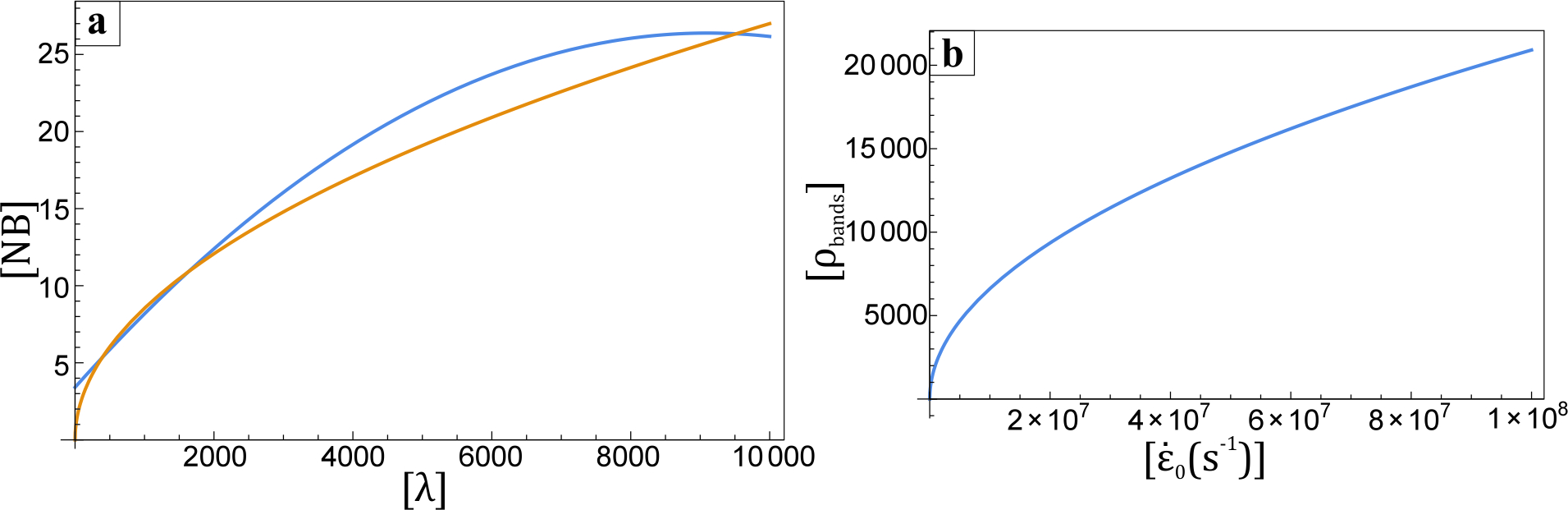


Figure S 2: a best fit of varying λ and the number of bands (NB). If realistic values are inserted for µ and Pe, these parameters do not affect the scaling of the bands (tested ranges were *µ*=[10^-10^, 10^-20^] and *Pe* = [0,1]). The best fit is obtained for *NB = 0.27*$\sqrt{\lambda}$. This value had already been found to represent the best fit by Veveakis et al., 2015^4^.

b inversion for the reference strain rate () and the band density per m (*ρ_bands_*) based on the best fit (fig.S2a).

The connection between band density and reference strain rate is shown in figure S2b. If the relationships in figure S2 are determined, the results can be displayed in a log-log plot that visualizes the relation between overpressure, permeability and the observable number of bands per meter (figure 2). Note that the parameters *Pe* and *μ* have inappreciable effect in the scaling depicted in figure S2, while the parameter α can influence it weakly (figure S3).

Figure S 3: Parametric study for the number of bands with respect to the α-parameter. The number of bands (NB) scales as *N_B = 0.27*$\sqrt{\lambda}$ for *α* = 1 (black line) and as *NB = 0.43*$\sqrt{\lambda}$ for *α* = 10 (red line). There is therefore a weak dependence of the scaling with$a$. In this example we used $Pe=0.1 , \mu=10^{-6} \lambda$ .

**Additional evidence from observations and discussion**

In figure S4 we present additional evidence for the mobility of second phase particles during recrystallization. We describe the recrystallization as a process of coupled dissolution precipitation^5,6^ on the atomic scale rather than. This process can release the second phase particles and collects them in the grain boundaries, which can be seen in thin-sections of white and dark bands of the pattern (fig. S4).


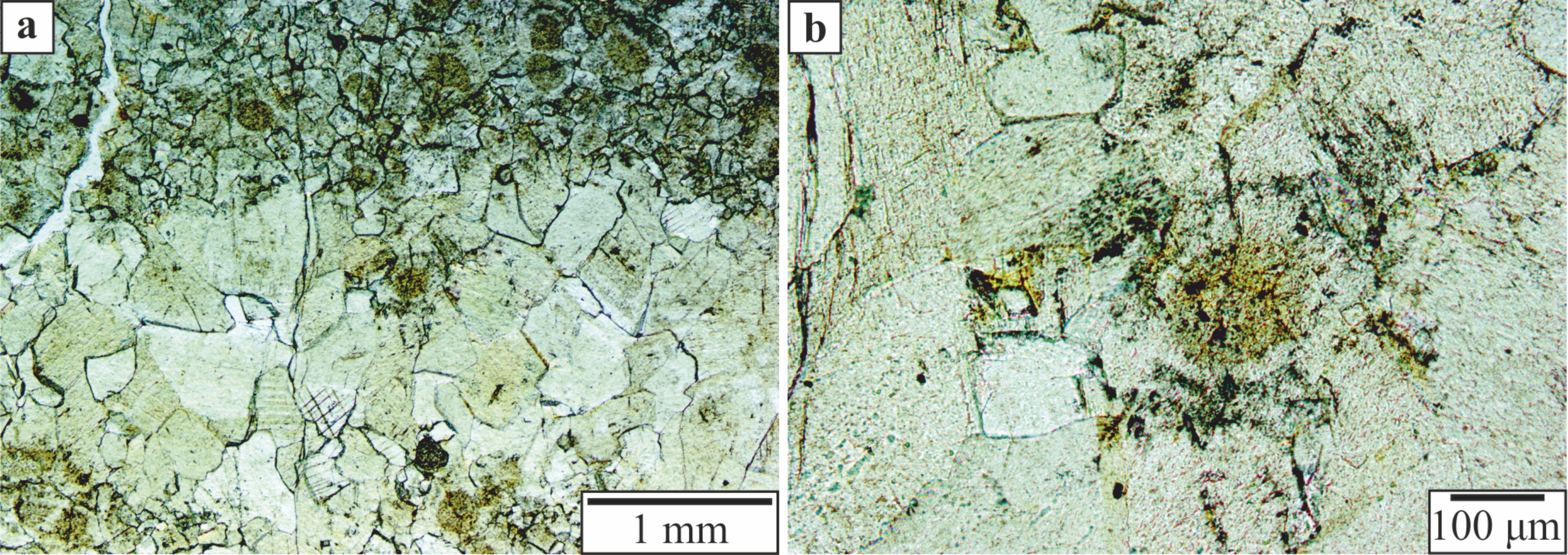


**Figure S 4**: Micrograph of zebra dolomite sample from the San Vicente mine, Peru. **a** Dark, fine-grained zebra dolomite layer (*AB_s_*) (top) in which numerous sedimentary features (Ooid-ghosts) are preserved. Grain boundaries in this layer appear very dark and the overall impurity density is conversably higher than in the light coarse-grained layer (*A_s_*) below. Note that crystals at the margin between the dark and light zebra band comprise of an impurity-rich nucleus that is located in the dark area and become impurity-free in the light layer.

**b** Detailed view of the Ooid-ghost within the light layer shown in **a**. The structure looks “torn apart” with impurities accumulating on grain boundaries around the structure.

The initial replacement reaction of limestone to dolomite leaves Ooid “ghosts” behind, which are original sedimentary structures. The second reaction, where dolomite is replaced by dolomite during grain growth moves the impurities around, because they are partly captured in the grain boundaries. The impurities appear as dark grain boundaries in the dark as well as the white bands and as rings around Ooids. The bi-modal crystal size in the white and dark bands is also explained by the abundance of impurities. If there are too many impurities in a layer the grain boundaries are pinned (Zener pinning) and grain growth cannot take place or is very slow. However, if impurity content is low grains can grow and they can also move the impurities around, collect them in grain boundaries and become clean.

One has to note that our model for the spacing of the pattern is different from other hypotheses on the zebra dolomite formation^7-9^ that need displacive veins that push the light layers apart by the force of crystallization. This induced stress is thought to produce the spacing of the layers, similar to stress shadows. Merino et al.^7-9^ argue that stylolites in the dark layers, which are very common in these rocks, are an indication for this induced stress. A problem with this argument is that stylolites would develop anyhow under these conditions and they would preferably grow in the dark bands with a large amount of impurities. We are of the opinion that our model is more general and can include and combine all observations and models of layered dolomites. In addition to that, our model does not need the induced stress, however, it also does not contradict the existence of induced stress in the growing bands. In addition our model can explain the large differences in layer spacing (orders of magnitude) that exist in Zebra dolomites in contrast to other models that would rather produce uniform spacing^7-9^.

Table S2: Indicative values used during the inversion. For these values the dimensionless groups of (S7) are calculated in the Mathematica script. It has to be noted that kinetic parameters for carbonates and especially for dolomite are poorly known. For example, the activation energy (*E*) exhibits uncertainties of up to 300%^10^.

| **Material Properties** | |
| --- | --- |
| Temperature, *T*^11^ | 70 - 160^○^ C |
| Burial depth, *y_0_*^11^ | 2- 3km |
| Kinematic viscosity^12^ | 0.4181 – 0.6191 (6 M NaCl, 35 MPa) |
| Dynamic viscosity^12^ | 480.5 - 726.3 μPas (6 M NaCl, 35 MPa) |
| Fluid density, ρ_f_ (calculated based on *T* and salinity): | 1250- 1279 kg/m^3^ |
| Dolomite density, *ρ_s_* | 2860 – 2930 kg/m^3^ |
| Dissolution rate of dolomite I, *A* (interpolation to pH 5)^13^ | 5·10^-7^ mol m^-2^s^-1^ |
| Activation energy dolomite, *E^14^* | 31.9 kcal mol^-1^ |
| Molar volume of dolomite, *v* | 63∙10^-6^ m^3^ mol^-1^ |
| Estimated volume change | ~1% |
| Average grain size, *d_0_* | 50 - 100 μm |
| Porosity of dolomite, *φ* | 1-5% |
| **Reference values for figure 2** | |
| Reference strain rate (): | 10^-5^ s^-1^ |
| Reference stress (*σ_0_*) | 5·10^9^ Pa |
| Yield stress (*σ_y_*) | 5·10^7^ Pa |
| Reference position (*y_0_*) | 2000 m |

**Remark:** The governing Equation (S7) was solved using the script of the following pages (also uploaded online) in Mathematica 10.3.0.0

**References**

1 Alevizos, S. *et al.* A Framework for Fracture Network Formation in Overpressurised Impermeable Shale: Deformability Versus Diagenesis. *Rock Mechanics and Rock Engineering*, 1-15, doi:10.1007/s00603-016-0996-y (2016).

2 Veveakis, E. & Regenauer-Lieb, K. Cnoidal waves in solids. *J Mech Phys Solids* **78**, 231-248, doi:10.1016/j.jmps.2015.02.010 (2015).

3 Karato, S. I. Deformation of Earth Materials: An Introduction to the Rheology of Solid Earth. *Deformation of Earth Materials: An Introduction to the Rheology of Solid Earth*, 347-349, doi:10.1017/Cbo9780511804892 (2008).

4 Veveakis, E., Regenauer-Lieb, K. & Weinberg, R. F. Ductile compaction of partially molten rocks: the effect of non-linear viscous rheology on instability and segregation. *Geophys J Int* **200**, 519-523, doi:10.1093/gji/ggu412 (2015).

5 Putnis, C. V., Ruiz-Agudo, E. & Hövelmann, J. Coupled fluctuations in element release during dolomite dissolution. *Mineralogical Magazine* **78**, 1355-1362, doi:10.1180/minmag.2014.078.6.01 (2014).

6 Putnis, A. Mineral replacement reactions: from macroscopic observations to microscopic mechanisms. *Mineralogical Magazine* **66**, 689-708, doi:10.1180/0026461026650056 (2002).

7 Merino, E. Self-accelerating dolomite-for-calcite replacement and displacive zebra veins: Dynamics of burial dolomitization. *Geochim Cosmochim Ac* **70**, A417-A417, doi:10.1016/j.gca.2006.06.839 (2006).

8 Merino, E. & Canals, À. Self-Accelerating Dolomite-for-Calcite Replacement: Self-Organized Dynamics of Burial Dolomitization and Associated Mineralization. *Am J Sci* **311**, 573-607, doi:10.2475/07.2011.01 (2011).

9 Merino, E., Canals, À. & Fletcher, R. C. Genesis of self-organized zebra textures in burial dolomites: Displacive veins, induced stress, and dolomitization. *Geol Acta* **4**, 383-393 (2006).

10 L'vov, B. V. *Thermal decomposition of solids and melts: new thermochemical approach to the mechanism, kinetics and methodology*. Vol. 7 209 (Springer Science & Business Media, 2007).

11 Fontboté, L. & Gorzawski, H. Genesis of the Mississippi Valley-Type Zn-Pb Deposit of San Vicente, Central Peru - Geologic and Isotopic (Sr, O, C, S, Pb) Evidence. *Econ Geol Bull Soc* **85**, 1402-1437 (1990).

12 Kestin, J., Khalifa, H. E. & Correia, R. J. Tables of the Dynamic and Kinematic Viscosity of Aqueous Nacl Solutions in the Temperature-Range 20-150-Degrees-C and the Pressure Range 0.1-35 Mpa. *J Phys Chem Ref Data* **10**, 71-87 (1981).

13 Gautelier, M., Oelkers, E. H. & Schott, J. An experimental study of dolomite dissolution rates as a function of pH from -0.5 to 5 and temperature from 25 to 80°C. *Chem Geol* **157**, 13-26, doi:Doi 10.1016/S0009-2541(98)00193-4 (1999).

14 Arvidson, R. S. & Mackenzie, F. T. The dolomite problem: Control of precipitation kinetics by temperature and saturation state. *Am J Sci* **299**, 257-288, doi:DOI 10.2475/ajs.299.4.257 (1999).
